# Liquisolid System and Liqui-Mass System Are Not the Same

**DOI:** 10.1208/s12249-020-01650-y

**Published:** 2020-03-16

**Authors:** Matthew Lam, Taravat Ghafourian, Ali Nokhodchi

**Affiliations:** Pharmaceutics Research Laboratory, School of Life Sciences, University of Sussex, Brighton, UK

**Keywords:** liquisolid technology, liqui-pellet technology, liqui-mass technology, liqui-mass system, liquisolid system

## Abstract

This commentary is written in response to Pezzini’s research group commentary, which claimed Liqui-Pellet and liquisolid pellet are not different. Despite some similarities, there are crucial differences separating these two technologies. Liqui-Pellet uses liqui-mass system (wet mass/paste admixture), and liquisolid pellet uses liquisolid system (flowable powder admixture). The understanding of the well-defined term ‘liquisolid system’ is crucial to understand what is and is not liquisolid formulation. Spireas, who is the inventor of liquisolid technology, clearly defined liquisolid system in his patent document and publications. Since his first publication in 1998, there are around 200 articles about liquisolid formulations (extracted from Scopus), and with no exception, every single one of them followed the original definition of liquisolid system. Liqui-Pellet does not use liquisolid system and so calling it the same as liquisolid pellet, which uses liquisolid system, is incorrect and misleading. The purpose of this commentary is to resolve misunderstanding and support furthering knowledge.

It has been shown that, although liquisolid technology is capable of enhancing or controlling the drug release rate, it seems the industrial application of this technique may be hampered due to a few hurdles such as low load factor, poor flowability and poor compactibility (particularly for high dose drugs). This was extensively discussed by Nokhodchi *et al.* in 2011 through a review article entitled ‘Drug release from liquisolid systems: speed it up, slow it down’ (1). It is obvious from Nokhodchi *et al*.’s review article that in order to overcome these hurdles, the flow of powders containing liquid medication requires improvement. It is also clear from Spireas’ patent (2) that liquisolid powders should be flowable by adding more carriers, which in turn reduces load factor and makes the final dosage form too bulky for commercial use. In order to have a high load factor, a wet mass system (non-flowable powder) should be produced which is different from liquisolid system (flowable powder). So, on the basis of this, there is a clear difference between ‘liquisolid pellet’ and ‘Liqui-Pellet’ in that liquisolid pellet uses ‘liquisolid system’ and Liqui-Pellet uses ‘liqui-mass system’. In the commentary written by Pezzini *et al*., it is mentioned that ‘liquisolid pellets (or liqui-pellets) are liquisolid system in nature’ and that the only difference is in the name (3). This statement is incorrect and misleading. To merely say liquisolid pellet and Liqui-Pellet are the same in nature implies a lack of fundamental and technical understanding of these different methodologies. Indeed, there are similarities, but there are also crucial differences.

To clarify the differences between liquisolid pellet and Liqui-Pellet, it is imperative to know what is considered a liquisolid formulation and what is the exact definition of a liquisolid system. In the patent document published by Spireas (2), who is the inventor of liquisolid technology, liquisolid system and liquisolid compact are clearly defined. Below is the exact definition taken from the Spireas patent document.

- The term ‘liquisolid systems’ refers to powdered forms of liquid medications formulated by converting liquid lipophilic drugs, or drug suspensions or solutions of water-insoluble solid drugs in suitable non-volatile solvent systems, into ‘dry’ (*i.e.*, dry-looking), non-adherent, free-flowing and readily compressible powder admixtures by blending with selected carrier and coating materials (2)
- The term ‘liquisolid compacts’ refers to immediate or sustained release tablets or capsules that are prepared using the technique described under ‘liquisolid systems’ (2)

Since Spireas’ first publication in 1998 entitled ‘*In vitro* evaluation of hydrocortisone liquisolid tablets’ (4), there are around 200 articles published in various pharmaceutical journals (extracted from Scopus), and with no exception, every single one of them followed the original definition of liquisolid system introduced by Spireas (dry-looking and free-flowing powder). So, it is quite common and acceptable now when any article uses the term ‘liquisolid’; it means that the resultant admixture is under liquisolid system, which is dry-looking and free-flowing. Therefore, it is clear that Liqui-Pellet does not fall under liquisolid system as the admixture of API and excipients are not ‘dry-looking’ and ‘non-adherent’, but in fact is a wet mass/paste even before introducing granulating liquid, which will eventually evaporate. Such a system is termed liqui-mass system, which is a fundamental difference between liquisolid technology and Liqui-Pellet technology (also referred to as Liqui-Mass technology). This is already explained in Lam *et al*.’s publications on Liqui-Pellet (5,6) and Lam *et al*.’s patent application (7). In this patent application, Lam *et al.* have also included some data on how effervescent Liquid-Pellet and sustained release Liquid-Pellet can be manufactured. In addition, the patent application shows the application of liqui-mass system in high-dose drug formulations such as ketoprofen and also explains the application of anti-tack agent in liqui-mass system.

Furthermore, Pezzini *et al.* claim ‘there are no differences in manufacturing and composition (besides model drug) between our first reported liquisolid pellet and the reported Liqui-Pellet by Lam *et al.*’ (3). Such a claim is surprising because not only is the system different but also the composition and drying method are clearly different, which is shown in Table I.

**Table I:** Differences between Liqui-Pellet and liquisolid pellet based on Lam *et al*.’s and Pezzini *et al*.’s publications (5,8)

Title of article	Liqui-Pellet: the emerging next-generation oral dosage form which stems from liquisolid concept in combination with pelletisation technology	Liquisolid technology applied to pellets: evaluation of the feasibility and dissolution performance using felodipine as a model drug
Dosage form name	Liqui-Pellet	Liquisolid pellet
System	Liqui-mass system	Liquisolid system
Liquid loading factor	1	0.1
Percentage of non-volatile solvent in total pellet mass	~ 29%	~ 5%
Granulating liquid	Deionised water	Copovidone in water (1%)
Drying method	Oven-drying	Fluid bed dryer
Coating material	Aerosil 300	Crospovidone(also disintegrant)

To avoid confusion and promote transparency, this commentary article will be approached in a chronological manner. Pezzini *et al.* published their first official article on liquisolid pellet in 2016 (8). Indeed, to our knowledge, they were the first to publish an article on liquisolid pellet, and the low amount of non-volatile co-solvent and a high amount of carrier material with a low liquid load factor of 0.1 is typical of liquisolid system. Throughout this article, ‘liquisolid system’ was consistently mentioned, and agreed upon is a dry-flowing powder. The phrases below are taken from Pezzini *et al.*’s own publication on liquisolid pellet to support our claim:

- ‘The liquid medication was incorporated in the microcrystalline cellulose 101, which acted as a carrier in the liquisolid system…’ (8)
- ‘Crospovidone was added to the powder mixtures to act as coating material for the liquisolid system, giving a dry aspect to the powder…’ (8)
- ‘…liquisolid systems (or liquisolid compacts) define them as acceptably flowing and compressible powdered forms of liquid medications’ (8)

Lam *et al.* have first worked on Liqui-Pellet in 2015 (this was Lam’s PhD thesis), but due to the confidential nature of the data obtained, the data was not published until a later date. There are formal records to support this claim. Later on, Liqui-Pellet was disclosed in a patent application in 2018 (9) followed by two publications in 2019 (5,6). The first two publications on Liqui-Pellet explains the fundamental difference between liquisolid system and liqui-mass system, which is crucial in giving Liqui-Pellet versatility and high liquid load factor, where ~ 38% of the total mass of pellet is non-volatile co-solvent (6).

In the same year of 2019, Pezzini *et al*. published a paper called ‘Liquisolid pellets: A pharmaceutical technology strategy to improve the dissolution rate of ritonavir’ (10). At this point, there seems to be a misunderstanding. What Pezzini *et al.* called liquisolid pellet resembles a formulation using liqui-mass system, which is disclosed in Lam *et al*.’s publications (5,8). Given the extensive use of the well-defined term ‘liquisolid system’, the dosage form Pezzini *et al*. mentioned in this particular publication cannot be described as liquisolid formulation as the admixture of API, and excipients is most likely a wet mass/paste rather than flowable powder that defines liquisolid system. This is due to the high amount of non-volatile co-solvent rendering the admixture non-flowable. In Pezzini *et al*.’s previous publication in 2016 (8), they did indeed make liquisolid pellet; however, their publication in 2019 (10) is not liquisolid pellet. What Pezzini *et al*. appear to believe to be liquisolid pellet cannot be described as liquisolid, because it does not fall under the well-defined liquisolid system. Since Pezzini *et al*. believe that they made liquisolid pellet without using liquisolid system, this gives rise to the confusion that liquisolid pellet and Liqui-Pellet are the same.

Please note that Pezzini *et al*. published a commentary in November 2019 (3), implying that Lam *et al*. did not recognise Pezzini *et al*.’s work on liquisolid pellet, ‘we believe in scientific cordiality and giving credit where credit is due’. Lam *et al*. published an article on Liqui-Pellet in July 2019 (6) mentioning Pezzini *et al*.’s work on liquisolid pellet as shown below:

- ‘Pezzini *et al*. have applied liquisolid system to pellet, but in the current study, liqui-mass system was used instead. Both liqui-pellet and liquisolid pellet do indeed contain inherent advantage from liquisolid and pelletisation technologies. However, they are distinctively different in that they both use different systems (i.e. liqui-mass system *versus* liquisolid system)’ (6)

We note that Pezzini *et al*. did not include this in their commentary article (3), despite the article being available as open access since July 2019. Nonetheless, Lam *et al*. acknowledged Pezzini’s work on liquisolid pellet.

In summary, there is a fundamental difference between liquisolid pellet and Liqui-Pellet. Liquisolid pellet uses liquisolid system and Liqui-Pellet uses liqui-mass system. To our knowledge, Pezzini *et al*. were the first to publish on liquisolid pellet, and Lam *et al*. are the first to publish on Liqui-Pellet. Liqui-Pellet is not considered liquisolid compact. Lam *et al*. acknowledge Pezzini *et al*.’s commentary and support dialogue to resolve misunderstanding and furthering knowledge.

## References

[1] Nokhodchi A, Hentzschel CM, Leopold CS (2011). Drug release from liquisolid systems: speed it up, slow it down. Expert Opin Drug Deliv.

[2] Spireas S. Liquisolid systems and methods of preparing same. 2002.

[3] Pezzini BR (2020). Liquisolid pellets and liqui-pellets are not different. AAPS PharmSciTech.

[4] Spireas S, Sadu S, Grover R (1998). In vitro evaluation of hydrocortisone liquisolid tablets. J Pharm Sci.

[5] Lam M, Ghafourian T, Nokhodchi A (2019). Liqui-pellet: the emerging next-generation oral dosage form which stems from liquisolid concept in combination with pelletization technology. AAPS PharmSciTech.

[6] Lam M, Ghafourian T, Nokhodchi A. Optimising the release rate of naproxen liqui-pellet: a new technology for emerging novel oral dosage form. Drug Deliv Transl Res. 2019:1–16. 10.1007/s13346-019-00659-6 PMC6978301 31286452

[7] Lam M, Nokhodchi A. International PCT patent GB2019/052065. 2019.

[8] Pezzini BR (2016). Liquisolid technology applied to pellets: evaluation of the feasibility and dissolution performance using felodipine as a model drug. Chem Eng Res Des.

[9] Lam M & Nokhodchi A. UK patent application, no. 1812022.0. 2018.

[10] De Espíndola B (2019). Liquisolid pellets: a pharmaceutical technology strategy to improve the dissolution rate of ritonavir. Saudi Pharm J.

